# Pretreatment HIV drug resistance in adults initiating antiretroviral therapy in China, 2017

**DOI:** 10.1186/s40249-020-00668-5

**Published:** 2020-05-24

**Authors:** Rui-Hua Kang, Shu-Jia Liang, Yan-Ling Ma, Shu Liang, Lin Xiao, Xin-Hui Zhang, Hong-Yan Lu, Xiao-Qin Xu, Shui-Bin Luo, Xiao-Guang Sun, Lin Chen, Jian-Mei He, Guo-Hui Wu, Ling-Jie Liao, Hui Xing, Yi-Ming Shao, Yu-Hua Ruan

**Affiliations:** 1 State Key Laboratory of Infectious Disease Prevention and Control (SKLID), National Center for AIDS/STD Control and Prevention (NCAIDS), Chinese Center for Disease Control and Prevention (China CDC), Collaborative Innovation Center for Diagnosis and Treatment of Infectious Diseases, 155 Changbai Road, Changping District, Beijing, PR China; 2 Guangxi Center for Disease Control and Prevention, Nanning, Guangxi China; 3 Yunnan Center for Disease Control and Prevention, Kunming, Yunnan China; 4 Sichuan Center for Disease Control and Prevention, Chengdu, Sichuan China; 5 Liangshan Center for Disease Control and Prevention, Liangshan prefecture, Sichuan China; 6 Guizhou Center for Disease Control and Prevention, Guiyang, Guizhou China; 7 Beijing Center for Disease Control and Prevention, Beijing, China; 8 Jiangsu Center for Disease Control and Prevention, Nanjing, Jiangsu China; 9 Neijiang Center for Disease Control and Prevention, Neijiang, Sichuan China; 10 Shandong Center for Disease Control and Prevention, Jinan, Shandong China; 11 Shenzhen Center for Disease Control and Prevention, Shenzhen, Guangdong China; 12 Hunan Center for Disease Control and Prevention, Changsha, Hunan China; 13 Chongqing Center for Disease Control and Prevention, Chongqing, China

**Keywords:** HIV, Pretreatment drug resistance, Transmission network, Antiretroviral therapy

## Abstract

**Background:**

After the scale-up of antiretroviral therapy (ART) for HIV infected people, increasing numbers of patients have pretreatment drug resistance (PDR). In this study, the prevalence of PDR was evaluated in adults initiating antiretroviral therapy in China.

**Methods:**

Blood samples were obtained from 1943 patients who initiated antiretroviral therapy (ART) in 2017 from 13 provinces or cities in China. Pol sequences were used to analyze drug resistance and construct transmission networks. Logistic regression model was used to estimate the potential factors associated with PDR.

**Results:**

In total, 1711 eligible patients (76.0% male; 87.8% aged ≥ 25 years) were included, of which 117 (6.8%) had PDR. The highest rates of PDR were 12.2% in Liangshan Prefecture of Sichuan and 9.3 and 8.9% in Dehong and Lincang Prefecture of Yunnan. A multivariate logistic regression analysis revealed that PDR was significantly higher among intravenous drug users (adjusted Odds Ratio (a*OR*) = 2.64, 95% *CI*: 1.57–4.44) and individuals from Liangshan, Dehong, and Lincang (a*OR* = 2.04, 95% *CI*: 1.26–3.30). In total, 754 sequences were used to generate 164 transmission networks. Five transmission networks had two or three sequences containing the same mutations, two networks contained subjects from Liangshan, and one network contained subjects from Dehong.

**Conclusions:**

Overall, the PDR prevalence was moderate, with a particularly high prevalence in areas with severe HIV epidemics. These results indicate the importance of continuous PDR monitoring in patients initiating antiretroviral therapy.

## Background

Highly active antiretroviral therapy has decreased mortality rates and prolonged the lives of people living with HIV or AIDS (PLWHA) [1–4]. In China, the nationwide National Free Antiretroviral Treatment Program (NFATP) has been ongoing for 14 years. With the NFATP development and the formulation of the UNAIDS targets of “90–90-90” by 2020 [5], and the most important to improve the quality of life for PLWHA, the number of people receiving antiretroviral therapy (ART) has increased. With the rapid scale-up of treatment, the transmission of drug-resistant viruses can become a challenge among those newly infected with HIV. Individuals infected with drug-resistant HIV strains may experience early virological failure [6–8], and the accumulation and transmission of drug-resistant strains can result in an increased mortality rate. Pretreatment drug resistance (PDR) to standard first-line ART is a challenge for HIV treatment. The WHO recommends transmitted drug resistance surveillance among recently infected antiretroviral-naïve populations of younger than 25 years old [9]. In 2014, the WHO proposed a global strategy for the surveillance and monitoring of HIV drug resistance, including the surveillance of PDR in populations initiating ART [10, 11]. In 2017, the WHO published new guidelines regarding the selection of antiretroviral drugs in response to high levels of PDR [12]. In particular, if the prevalence of PDR to non-nucleoside reverse-transcriptase inhibitors (NNRTIs) is ≥10% among individuals initiating first-line ART, excluding those with previous antiretroviral drug exposure, an alternative first-line ART regimen as a supplement should be urgently considered in accordance with the consolidated guidelines on the use of antiretroviral drugs established in 2016 [13].

In China, the NFATP was initiated at a nationwide scale in 2003 [14] and all individuals with HIV infections who provide consent can currently be treated [15]. However, genotyping was not performed for PLWHA before the initiation of ART in China. In this large cross-sectional study conducted in multiple provinces in China in 2017, the prevalence of PDR among all adults was evaluated. This study is to estimate the prevalence of PDR among individuals ≥18 years of age as well as the associated risk factors and to explore PDR related transmission in China.

## Methods

### Study design and study participants

This was a cross-sectional study to estimate the prevalence of PDR in adults initiating antiretroviral therapy in China. The method of HIVDR test was used to explore the level of PDR in part of regions in China. The study design was performed according to the 2014 WHO protocol for PDR [11]. China has twenty-three provinces, four municipalities, five autonomous regions, and two special administrative regions. HIV infection was originally identified among injected drug users (IDUs) in Yunnan Province, which is located in southwest China, and was subsequently disseminated into IDU populations in Guangxi Zhuang Autonomous Region and Sichuan Province [16–18]. HIV was subsequently disseminated by sexual transmission to populations in China. According to the numbers of newly reported HIV/AIDS in the year 2016 in China [19], the high prevalence regions were recognized when the number in the region was equal to 8000 or more; the moderate prevalence regions were defined when the number ranged from 2500 to 7999; and the low prevalence region refers to where the number was below 2500. This pilot investigation was conducted in high prevalence regions (Yunnan Province which included Dehong Prefecture and Lincang City, Guangxi Zhuang Autonomous Region which included Nanning City and Liuzhou City, Sichuan Province which included Liangshan Prefecture and Neijiang City, Guangdong Province which included Shenzhen City) and moderate prevalence regions (Chongqing Municipality, Guizhou Province, Hunan Province, Beijing Municipality, Jiangsu Province, and Shandong Province). WHO recommended that the number of patients would typically fall within the range of 300–500 during 6 months in a country according to the HIV drug resistance surveillance guideline in 2015 [20]. According to this guideline, at least 300 patients were recruited in high prevalence regions and moderate prevalence regions.

The patients’ recruitment and specimens collection were performed from January 2017 to June 2017 (6 months). Two clinics were randomly selected in each study area. Patients were recruited at the selected clinics during the study period depending on the enrollment order. And the recruitment was based on sequential sampling of patients who received ART treatment at the clinics [20]. All recruited patients were at age 18 or older and initiated ART after this investigation. Excluded from this study were patients who were under 18 years old, or those who may be acquired drug resistance from previous antiretroviral drug exposure (such as among women exposed to ARV drugs for the prevention of mother-to-child transmission (PMTCT) of HIV, among individuals reinitiating first-line ART after a period of treatment interruption without documented viral failure or among people who have received pre-exposure prophylaxis (PrEP). All patients provided written informed consent.

### Data collection

Baseline variables were obtained for all patients, including socio-demographic and behavioural characteristics. The variables of date of birth, sex, marital status, ethnicity, years of education and route of HIV infection were achieved from the national epidemiology database of HIV infection [21]. History of ART drug use was asked by the doctor and validated by the NFATP database of HIV infection [22]. To avoid the patients with a history of ART were recruited, the doctor would reinvestigate these patients by interviewing or phone, especially these with PDR. CD4 cell counts before ART were tested within 12 h after sample collection and sampling region was the site of sampling clinics.

### Laboratory tests

Whole blood was sampled before the initiation of ART. CD4 cells were counted by flow cytometry at local CDCs within 12 h of sample collection. Plasma was isolated and sent under cold-chain conditions to the laboratory at NCAIDS, China CDC. RNA was extracted from 200 μl of plasma, amplified, and used to sequence the HIV *pol* region following an in-house method [23, 24]. The definition of any drug resistance is defined with respect to one or more of the following drugs or drug classes: Efavirenz (EFV), Nevirapine (NVP), any NRTI, Darunavir (DRV/r), Lopinavir (LPV/r), or Atazanavir (ATV/r) [11]. The classification “susceptible or potential low-level” indicates no drug resistance (Stanford penalty score < 15) and a classification of at least low-level indicates drug resistance (Stanford penalty score ≥ 15) [11]. PDR was evaluated using the algorithm of the Stanford HIV Drug Resistance Database (HIVDB) (https://hivdb.stanford.edu/hivdb/by-sequences/).

### Identification of genetic transmission networks

To avoid potential biases due to convergent evolution, 43 codons in *PR* and *RT* associated with drug resistance mutations were removed according to the most recent update of major HIV-1 drug resistance mutations [25]. Finally, the sequences were 910 bp in length. To construct genetic transmission networks, the pairwise Tamura–Nei 93 (TN93) genetic distances [26, 27] were calculated among all sequences using HYPHY2.2.4. Transmission networks were visualized and analyzed using Cytoscape3.5.1 with a threshold genetic distance of 0.0125 among subtypes. The genetic distance threshold was defined as the distance that identifies the maximum number of clusters in the transmission networks [27]. The cluster included three or more same drug resistance mutations (DRMs) was defined as the HIV drug resistance (HIVDR) related cluster. The DRMs in the same clusters may be transmitted potentially. To visualize the networks, the igraph and network packages in R 3.5.0 software (the Free Software Foundation’s GNU General Public License, Auckland, New Zealand) were used [28].

### Statistical analysis

In this cross-sectional study, we collected baseline data and obtained subtypes after sequencing analysis of participants. Univariate and multivariate logistic regression models were used to estimate the potential factors associated with pretreatment drug resistance. We adjusted age, sex, marital status, ethnicity, year of education, route of infection, CD4 cell counts before ART, subtype, and region for each participant. We constructed multivariate logistic regression model in a stepwise manner to select variables independently associated with drug resistance. Odds ratio (*OR*) and adjusted odds ratio (a*OR*) with 95% confidence interval (95% *CI*) were reported. All tests were two tailed and a *P*-value < 0.05 was considered statistically significant. Statistical analyses were performed using SAS V9.4 (SAS Institute Inc., Cary, NC, USA).

## Results

### General characteristics of the study population

During the study period, we recruited 1943 participants in total and acquired 1731 (89.1%) sequences. Twenty participants were excluded, including fourteen participants who were aged below 18 years and six without any information. Finally, 1711 participants were included in the study (Table 1). In total, 87.8% (1502/1711) of participants were ≥ 25 years old and 76.0% (1301/1711) were male. The numbers of unmarried, married, and divorced or widowed participants were 562, 873, and 271, respectively (32.8, 51.0, and 15.8%). Marital status was missing for five patients (0.3%). The majority of participants (65.9%, 1128/1711) were of Han ethnicity and 66.3% received a junior high school-level education or below. More than half (60.0%) of participants were infected via heterosexual intercourse, followed by homosexual intercourse (26.5%) and IDUs (9.4%). In total, 1076 (62.9%) patients had CD4 cell counts < 350 cells/ml and 576 (33.7%) had CD4 cell counts ≥350 cells/ml. In addition, no records of CD4 cell counts were obtained for 59 patients. The proportions of the subtypes CRF01_AE, CRF07_BC, CRF08_BC, CRF55_01B, and others were 32.7% (559/1711), 37.0% (633/1711), 13.3% (228/1711), 3.7(64/1711), and 7.8% (134/1711), respectively. Participants were primarily from Southwest China (52.0%, 889/1711). None of the participants had previous exposure to antiretroviral drugs.

**Table 1:** Characteristics of HIV-infected individuals initiating ART in 2017 in China

Variable	Number	Percentage (%)
Total	1711	100.0
Age (years)		
< 25	209	12.2
≥ 25	1502	87.8
Sex		
Male	1301	76.0
Female	410	24.0
Marital status		
Unmarried	562	32.8
Married	873	51.0
Divorce or widow	271	15.8
Unknown	5	0.3
Ethnic		
Han	1128	65.9
Others	583	34.1
Education		
Primary and below	638	37.3
Junior high school	496	29.0
Senior high school	217	12.7
College	307	17.9
Unknown	53	3.1
Route of infection		
Heterosexual intercourse	1027	60.0
Homosexual intercourse	453	26.5
Intravenous drug use	160	9.4
Other	71	4.1
CD4 count before ART (cells/mm^3^)		
< 350	1076	62.9
≥ 350	576	33.7
Missing	59	3.4
Subtype		
CRF01_AE	559	32.7
CRF07_BC	633	37.0
CRF08_BC	228	13.3
CRF55_01B	64	3.7
B	40	2.3
C	53	3.1
Others	134	7.8

### Pretreatment HIV drug resistance

In total 6.8% (117/1711) of patients had PDR. Among them, 79 (4.6%) had PDR to NNRTI, 38 (2.2%) to NRTI, and 10 (0.6%) to PI (Table 2). Seventy-six and seventy-nine participants had resistance to EFV and NVP, respectively. *K103N* (36, 2.1%), *V179D* (28, 1.6%), and *E138A* (18, 1.1%) were the most common mutations in the reverse transcriptase (RT) region. For NRTIs, the most frequent PDR drug was D4T (32, 1.9%), followed by AZT (20, 1.2%). The most frequent mutations were *K65R* (9, 0.5%) and *D67N* (8, 0.5%) in the RT region. All 10 patients with PDR to PIs were resistant to LPV/r and the most common mutations were *I47V* (3, 0.2%), *I50V* (2, 0.1%), *I54V* (2, 0.1%), and *V82A/L/F* (2, 0.1%) in the protease (PR) region.

**Table 2:** Pretreatment HIV drug resistance mutations among HIV-infected individuals with drug resistance

	*N* (%)	HIV drug resistance mutations, *N* (%)
Total	117 (6.8)	
Non-nucleoside reverse transcriptase inhibitors (NNRTIs, any)	79 (4.6)	
		K103N/S/T,36 (2.1)
		V179D/E/T,28 (1.6)
		E138AA/K/G/Q,18 (1.1)
		V106M,9 (0.5)
		G190A/S,6 (0.4)
		Y188L/C/F,6 (0.4)
Efavirenz (EFV)^a^	76 (4.4)	K101E,4 (0.2)
Nevirapine (NVP)^a^	79 (4.6)	Y181C,3 (0.2)
		H221Y,3 (0.2)
		A98G,2 (0.1)
		F227L,1 (0.06)
		L100I,1 (0.06)
		P225,1 (0.06)
Nucleoside reverse transcriptase inhibitors (NRTIs, any)	38 (2.2)	
		K65R,9 (0.5)
		D67N,8 (0.5)
		M184I/V,7 (0.4)
Emtricitabine (FTC)	12 (0.7)	T215D/I/S,7 (0.4)
Lamivudine (3TC)^a^	12 (0.7)	K70E/R/T,5 (0.3)
Abacavir (ABC)^a^	16 (0.9)	L210W,4 (0.2)
Didanosine (DDI)^a^	16 (0.9)	K219R,2 (0.1)
Stavudine (D4T)^a^	32 (1.9)	L74LI,2 (0.1)
Tenofovir (TDF)^a^	12 (0.7)	M41L,2 (0.1)
Azidothymidine (AZT)^a^	20 (1.2)	T69D,2 (0.1)
		Y115F,2 (0.1)
		A62V,1 (0.06)
		V75I,1 (0.06)
Protease inhibitors (PIs, any)	10 (0.6)	
		I47V,3 (0.2)
		I50V,2 (0.1)
Atazanavir (ATV)	5 (0.3)	I54V,2 (0.1)
Darunavir (DRV)	4 (0.2)	V82A/FI/L,2 (0.1)
Lopinavir (LPV)^a^	10 (0.6)	I84V,1 (0.06)
		L90M,1 (0.06)
		M46I/L,1 (0.06)
Multi-drug resistance to NNRTI and NRTI	6 (0.4)	
Multi-drug resistance to NNRTI, NRTI and PI	1 (0.06)	

^a^ National free antiviral drugs in China

*HIV* Human immunodeficiency virus, *NNRTI* Non-nucleoside reverse transcriptase inhibitor, *NRTI* nucleoside reverse transcriptase inhibitor, *PI* Protease inhibitor

The prevalence of PDR varied from different regions (Table 3). The overall PDR prevalence for all regions was 6.8% (117/1711). The most severely affected drug class was NNRTI (4.6%, 79/1711), followed by NRTI (2.2%, 38/1711) and PI (0.6%, 10/1711). Liangshan prefecture of Sichuan province had the highest PDR prevalence (12.2%, 34/279) among all regions, followed by Dehong prefecture (9.3%, 14/150) and Lincang prefecture (8.9%, 14/158) of Yunnan province.

**Table 3:** Pretreatment HIV drug resistance among HIV-infected individuals initiating ART in 2017 in China, by region

	Number	Number of PDR	Prevalence (%)	Prevalence (%)		
				NNRTIs	NRTIs	PIs
Total	1711	117	6.8	4.6(79/1711)	2.2 (38/1711)	0.6 (10/1711)
High prevalence regions	1102	90	8.2	5.5(61/1102)	2.8 (31/1102)	0.6 (7//1102)
Dehong, Yunnan	150	14	9.3	8.7(13/150)	0.7 (1/150)	0
Lincang, Yunnan	158	14	8.9	5.7(9/158)	3.8 (6/158)	1.3 (2/158)
Liuzhou, Guangxi	207	11	5.3	2.9(6/207)	1.9 (4/207)	0.5 (1/207)
Nanning, Guangxi	104	7	6.7	3.9(4/104)	3.8 (4/104)	0
Liangshan, Sichuan	279	34	12.2	9.0(25/279)	3.9 (11/279)	1.1 (3/279)
Neijiang, Sichuan	105	5	4.8	1.0(1/105)	2.9 (3/105)	1.0 (1/105)
Shenzhen, Guangdong	99	5	5.1	3.0(3/99)	2.0 (2/99)	0
Moderate prevalence regions	609	27	4.4	3.0(18/609)	1.1 (7/609)	0.5 (3/609)
Chongqing	85	5	5.9	2.4(2/85)	1.2 (1/85)	2.4 (2/85)
Guizhou	112	5	4.5	1.8(2/112)	1.8 (2/112)	0.9 (1/112)
Hunan	97	2	2.1	2.1(2/97)	1.0 (1/97)	0
Beijing	111	4	3.6	2.7(3/111)	0.9 (1/111)	0
Jiangsu	105	5	4.8	4.8(5/105)	0	0
Shandong	99	6	6.1	4.0(4/99)	2.0 (2/99)	0

*PDR* Pretreatment drug resistance, *NNRTI* Non-nucleoside reverse transcriptase inhibitor, *NRTI* nucleoside reverse transcriptase inhibitor, *PI* Protease inhibitor.

### Factors associated with HIV PDR

Risk factors associated with HIV PDR are listed in Table 4. In a univariate logistic regression analysis, four factors were significantly associated with HIV PDR. The *OR* for patients infected via IDU versus heterosexual intercourse was 3.61 (95% *CI*: 2.22–5.88) and that for Liangshan, Dehong, and Lincang versus other regions was 2.30 (95% *CI*: 1.57–3.35). A multivariate logistic regression model showed that IDUs and region (Liangshan, Dehong, and Lincang) were important factors, with a*OR*s of 2.64 (95% *CI:* 1.57–4.44) and 2.04 (95% *CI*: 1.26–3.30), respectively.

**Table 4:** Factors associated with pretreatment HIV drug resistance among HIV-infected individuals initiating ART in 2017 in China

Variable	Number	PDR, N (%)	*OR* (95% *CI*)	*P*-value	a*OR* (95% *CI*)	*P*-value
Total	1711	117 (6.8)				
Age (years)						
< 25	209	16 (7.7)	1.00			
≥ 25	1502	101 (6.7)	0.87 (0.50–1.51)	0.62		
Sex						
Male	1301	93 (7.2)	1.00			
Female	410	24 (5.9)	0.76 (0.48–1.22)	0.26		
Marital status						
Unmarried	562	38 (6.8)	1.00			
Married	873	59 (6.8)	1.00 (0.66–1.53)	0.99		
Divorce or widow	271	20 (7.4)	1.10 (0.63–1.93)	0.74		
Unknown	5	0 (0.0)	-	0.99		
Education						
Primary and below	638	52 (8.2)	1.00			
Junior high school	496	35 (7.1)	0.86 (0.55–1.34)	0.49		
Senior high school	217	14 (6.5)	0.78 (0.42–1.43)	0.42		
College	307	15 (4.9)	0.58 (0.32–1.05)	0.07		
Unknown	53	1 (1.9)	0.22 (0.03–1.60)	0.13		
Route of infection						
Heterosexual intercourse	1027	57 (5.6)	1.00		1.00	
Homosexual intercourse	453	27 (6.0)	1.08 (0.67–1.73)	0.75	1.48 (0.87–2.52)	0.15
Intravenous drug use	160	28 (17.5)	3.61 (2.22–5.88)	< 0.001	2.64 (1.57–4.44)	< 0.001
Others	71	5 (7.0)	1.29 (0.50–3.33)	0.60	1.56 (0.60–4.08)	0.36
CD4 count before ART (cells/mm^3^)						
< 350	1076	71 (6.6)	1.00			
≥ 350	576	45 (7.8)	1.20(0.81–1.77)	0.36		
Missing	59	1 (1.7)	0.24(0.03–1.79)	0.17		
Subtype						
CRF01_AE	559	26 (4.7)	1.00			
CRF07_BC	633	49 (7.7)	1.72 (1.05–2.81)	0.03		
CRF08_BC	228	19 (8.3)	1.86 (1.01–3.44)	0.05		
CRF55_01B	64	8 (12.5)	2.93 (1.27–6.78)	0.01		
B	40	1 (2.5)	0.53 (0.07–3.98)	0.53		
C	53	5 (9.4)	2.14 (0.78–5.81)	0.14		
Others	134	9 (6.7)	1.48 (0.68–3.23)	0.33		
Region						
Others	1124	55 (4.9)	1.00		1.00	
Liangshan, Dehong and Lincang^a^	587	62 (10.6)	2.30 (1.57–3.35)	< 0.001	2.04 (1.26–3.30)	0.004

^a^: The HIV epidemic was the most severe in Liangshan, Dehong and Lincang. The HIVDR rate was high in these areas

*PDR* pretreatment drug resistance, *OR* odds ratio, a*OR* adjusted odds ratio, *CI* confidence interval, *ART* antiretroviral therapy, *HIV* Human immunodeficiency virus, *HIVDR* HIV drug resistance; -: Not applicable.

### Genetic transmission networks

**Fig. 1:**
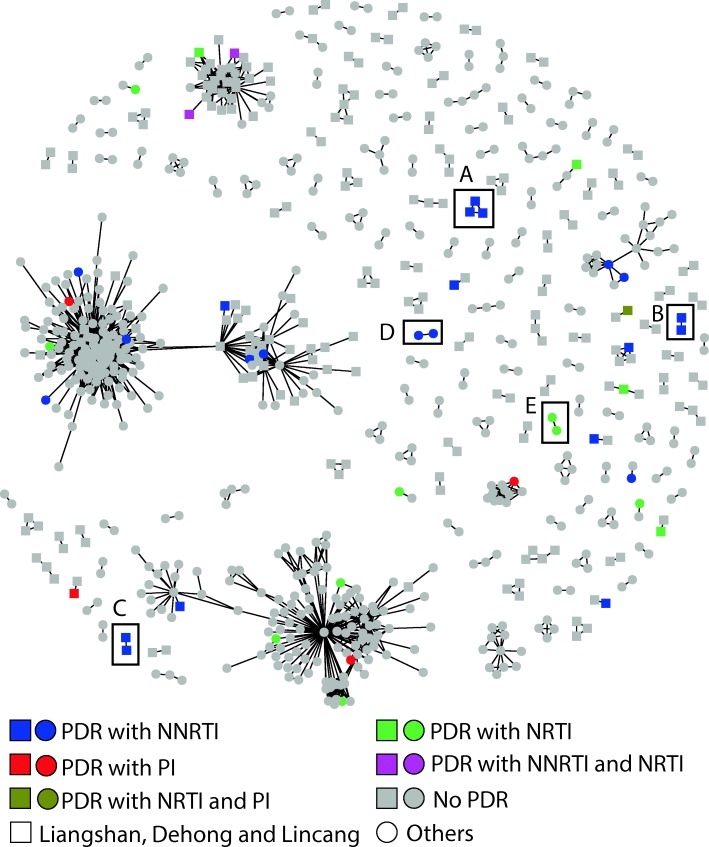
Pretreatment HIV drug resistance related transmission networks. Note: **a** Three sequences were from Liangshan and drug resistance mutations were *E138Q* and *V179D*. **b** Two sequences were from Liangshan and drug resistance mutation was *K103N*. **c** Two sequences were from Dehong and drug resistance mutations were *K103N* and *V106M*. **d** Two sequences were from Liuzou and drug resistance mutation was *K103N*. **e** Two sequences were from Shandong and drug resistance mutation was *L210W*. PDR: pretreatment drug resistance; NNRTI: non-nucleoside reverse-transcriptase inhibitor; NRTI: Nucleoside reverse-transcriptase inhibitor; PI: Protease inhibitor

In total, 1711 *pol* sequences were obtained to construct genetic transmission networks. We constructed transmission networks with a genetic distance threshold of 0.0125, which identified the maximum number of clusters. In total, 754 (44.1%) sequences were used to generate 164 transmission networks (Fig. 1). Among all transmission networks, 70.1% (115 of 164) included 2 individuals.

Overall, 43 sequences with PDR were included in the networks and the degrees between PDR and no PDR in the networks were not statistically significant. The 43 sequences with PDR were dispersed among 23 transmission networks; shared mutations yielded five transmission networks (Fig. 1). The most frequent DRMs in the transmission networks was *K103N* (7.9%, 13/164). Three transmission networks contained two PDR sequences with the *K103N* mutation and they were from Liangshan prefecture of Sichuan province, Dehong Prefecture of Yunnan Province, and Liuzhou City of Guangxi Province. In addition, three PDR sequences with *E138Q* and *V179D* mutations comprised a transmission network and they were from Liangshan prefecture of Sichuan province. Another transmission network included two PDR sequences with the *L210W* mutation from Shandong Province.

Among the 754 individuals included in the transmission networks, 49 were infected via IDU. Among these 49 individuals, five had PDR in five different networks. No transmission of the virus with PDR was observed among individuals who inject drugs.

## Discussion

We performed a cross-sectional study of HIV PDR in China and obtained an overall PDR prevalence of 6.8% (95% *CI*: 6.5–7.1%). According to the WHO, low, moderate, and high levels of HIV drug resistance are defined as < 5%, 5–15%, and > 15% [10]. PDR prevalence was moderate in China in 2017. The pooled prevalence of PDR was similar or higher than those for other countries, such as Zimbabwe (6.3%) [29] and Sub-Saharan Africa (5.5%) [30] but lower than estimates for Kenya (9.4%) [27], Argentina (14.0%) [31], and Mexico (15.5%) [32]. The prevalence of NNRTI PDR was 4.6% (95% *CI*: 4.4–4.8%) in this study, which is below the 10% threshold to change the recommended first-line ART regimen [12]. However, six countries have reported rates of PDR to NNRTI of > 10% [33]. Studies have suggested that PDR is associated with a shorter time to virological failure or increased risk of ART regimen switch [7, 30, 32]. Moreover, the most common mutation in this study was K103N, consistent with studies in Argentina, Zimbabwe, and Kenya [29, 31, 34]. Hence, routine surveillance programs for PDR in China are necessary.

In this study, there were no associations between age, sex, or CD4 cell counts before ART and PDR. These results were consistent with previous results in Vietnam and Zimbabwe [29, 35]. However, IDU was significantly associated with PDR, as observed in a previous study in Vietnam [29]. Another study has shown that IDUs receiving first-line ART have a higher rate of virological failure in China [36]. Although the overall prevalence of PDR in our study was moderate, the prevalence was high in some regions, i.e., Liangshan Prefecture of Sichuan, in Dehong Prefecture, and Lincang of Yunnan. Liangshan prefecture of Sichuan and Dehong prefecture of Yunnan are areas with serious HIV epidemics and IDUs are the main high-risk population for HIV infection. Antiretroviral treatment was initiated early and the highest rates of HIV-infected people receiving ART are in Sichuan and Yunnan.

In China, the recommended first-line regimen was zidovudine (AZT) or stavudine (D4T) + lamivudine (3TC) + nevirapine (NVP) in 2005 [22] and D4T was gradually replaced by AZT or tenofovir (TDF) from 2010 [37]. The current first-line regimen was TDF or AZT + 3TC + efavirenz (EFV) or NVP [15]. This study indicated that the HIVDR was high in the high prevalence regions, even though these patients without antiretroviral drugs exposure. The most common DRMs were related these drugs being used in China. Hence, the DRMs in treatment-naïve patients may originate from treatment failure.

Transmission networks were constructed to explore whether the HIVDR related cluster was existed among PLWHA before the initiation of ART. There were no significant differences between the degrees of PDR and no PDR. However, there were five transmission networks with two or three same DRMs. Two transmission networks contained subjects from Liangshan, and one contained subjects from Dehong. Another contained subjects from Liuzhou. All these cities are in regions with the most severe HIV epidemics. One additional transmission network contained subjects from Shandong, with moderate HIV rates. There was a HIVDR related cluster contained three same DRMs in Liangshan, especially. These results suggest that PDR could be transmitted among high-risk populations for HIV infection in the future.

The study had the following limitations. First, China has seven regions (northeast, north, northwest, central, east, south, and southwest). This pilot study survey did not include all these regions and was not representative of all individuals living with HIV or AIDS in China. We will expand the PDR study to all provinces in inland China next year. Second, the lack of drug-resistant virus transmission among IDUs in this study could be related to the sampling density. IDUs were not infected in local areas or related individuals were not recruited in this study.

## Conclusions

The present results demonstrate that the overall prevalence of PDR is moderate in China. However, in Liangshan prefecture of Sichuan, it exceeds 10%. There was no ongoing transmission of resistance-associated mutations in the networks. However, regional differences and complex epidemic trends suggest that the continuous surveillance of PDR is essential for estimating the level of PDR and ensuring the effectiveness of current ART regimens in China.

## Data Availability

The datasets are available from the corresponding author on reasonable request.

## Abbreviations

HIV: Human immunodeficiency virus
PDR: Pretreatment drug resistance
PLWHA: People living with HIV or AIDS
UNAIDS: United Nations programme on HIV/AIDS
WHO: World Health Organization
ART: Antiretroviral therapy
ARV: Antiretroviral virus
NFATP: National Free Antiretroviral Treatment Program
NNRTI: Non-nucleoside reverse-transcriptase inhibitor
NRTI: Nucleoside reverse-transcriptase inhibitor
PI: Protease inhibitor
IDUs: Injected drug users
PMTCT: Prevention of mother-to-child transmission
PrEP: Pre-exposure prophylaxis
EFV: Efavirenz
NVP: Nevirapine
FTC: Emtricitabine
3TC: Lamivudine
ABC: Abacavir
DDI: Didanosine
D4T: Stavudine
TDF: Tenofovir
AZT: Azidothymidine
DRV/r: Darunavir
LPV/r: Lopinavir
ATV/r: Atazanavir
DRM: Drug resistance mutation
HIVDR: HIV Drug Resistance Database
TN93: Tamura–Nei 93
*OR*: Odds ratio
a*OR*: Adjusted odds ratio
*CI*: Confidence interval
